# Outbreak by Ventilator-Associated ST11 *K. pneumoniae* with Co-production of CTX-M-24 and KPC-2 in a SICU of a Tertiary Teaching Hospital in Central China

**DOI:** 10.3389/fmicb.2016.01190

**Published:** 2016-08-02

**Authors:** Longhua Hu, Yanling Liu, Linqiang Deng, Qiaoshi Zhong, Yaping Hang, Zengzeng Wang, Lingling Zhan, Liangxing Wang, Fangyou Yu

**Affiliations:** ^1^Department of Clinical Laboratory, The Second Affiliated Hospital of Nanchang University, NanchangChina; ^2^Department of Clinical Laboratory, Jiangxi Provincial People’s Hospital, NanchangChina; ^3^Department of Laboratory Medicine, The First Affiliated Hospital of Wenzhou Medical University, WenzhouChina; ^4^Department of Respiratory Medicine, The First Affiliated Hospital of Wenzhou Medical University, WenzhouChina

**Keywords:** *K. pneumoniae*, carbapenemase, KPC-2, outbreak, ST11

## Abstract

The emergence of carbapenem-resistant *Klebsiella pneumoniae* (CRKP) often responsible for numerous hospital-associated outbreaks has become an important public health problem. From January 2013 to February 2014, a total of 41 non-duplicate *K. pneumoniae* isolates with carbapenem resistance, were collected at a tertiary teaching hospital in Nanchang, central China. Among 41 *K. pneumoniae* isolates, 28 were isolated from hospitalized patients including 19 from the patients in surgery intensive care unit (SICU) and 13 were isolated from ventilators. Twenty-four of 28 patients infected by CRKP have been submitted to mechanical ventilation using ventilator. More than 95% of the CRKP isolates were resistant to 13 antimicrobials tested. All CRKP isolates were confirmed as carbapenemase producer and were positive for *bla*_KPC-2_, with one positive for both *bla_KPC-2_* and *bla*_NDM-1_. All carbapenemase-producing isolates harbored at least one of extended spectrum β-lactamase genes tested, among which 95.1% (39/41) of the tested isolates were found to harbor both *bla*_CTX-M-24_ and *bla*_KPC-2_, Of note, one isolate harbored simultaneously two carbapenemase genes (*bla*_KPC-2_ and *bla*_NDM-1_) and two ESBL genes (*bla*_CTX-M-3_ and *bla*_TEM-104_). To the best of our knowledge, coexistence of *bla*_KPC-2_ and *bla*_CTX-M-24_ in one isolate is first reported. MLST results showed that 41 CRKP isolates belonged to four sequence types (STs) including ST11, novel ST1854, novel ST1855, and ST1224. PFGE results displayed three PFGE clusters. Thirty-eight ST11 CRKP isolates (92.7%, 38/41) including all 13 isolates from ventilators and 25 isolates from patients from seven wards (18 from SICU) belonged to same PFGE cluster, indicating these isolates were clonally related. Fifteen isolates have an identical undistinguished pattern (100% similarity) forming a single clonal population. Moreover, this clone was exclusively linked to the cases attended in SICU and linked to the Ventilators. Additionally, the other SICU cases were linked to closely related clones (similarity greater than 95%). These data indicated that the occurrence of a clonal outbreak associated with ventilators has been found. In conclusion, outbreak by ventilator-associated ST11 *K. pneumoniae* with co-production of CTX-M-24 and KPC-2 is found in a SICU of a tertiary teaching hospital in central China.

## Introduction

*Klebsiella pneumoniae* is an opportunistic pathogen associated mainly with pneumonia, bloodstream infections, and urinary tract infections. *K. pneumoniae* frequently exhibits multi-resistance to antimicrobial agents (Bratu et al., 2005). Carbapenems are often used for the treatment of clinical infections caused by this clinically important pathogen with multi-resistance to antimicrobial agents. However, the resistance of *K. pneumoniae* to carbapenems has been increasing (Gupta et al., 2011). As frequently carry additional antimicrobial resistance genes, *K. pneumoniae* isolates with carbapenem resistance often exhibit resistance to other non-β-lactam antimicrobial agents (Temkin et al., 2014). The emergence of carbapenem-resistant *K. pneumoniae* has become an important public health problem. The mechanism of resistance to carbapenems is mainly associated with production of carbapenemases (Nordmann and Poirel, 2002). Acquired carbapenemases encoded by transferable genes located in mobile elements including plasmids and transposons disseminate rapidly among different strains and species among *Enterobacteriaceae* (Temkin et al., 2014). *K. pneumoniae* carbapenemases (KPCs) and class B metallo-β-lactamases (MBLs) are found to be main carbapenemases in *K. pneumoniae*. In China, KPC-2 is a predominant carbapenemase and has been widely recognized in many species of *Enterobacteriaceae* including *K. pneumoniae, Serratia marcescens*, *Escherichia coli*, and *Enterobacter cloacae* (Cai et al., 2008; Wu et al., 2010). In 2009, a novel MBL, New Delhi metallo-lactamase 1 (NDM-1) initially identified in a *K. pneumoniae* isolate represents a new challenge for the treatment of infectious diseases (Yong et al., 2009; Kumarasamy et al., 2010). Dissemination of KPC-2 producing *K. pneumoniae* has been found in many Chinese hospitals (Yang et al., 2013; Hu et al., 2014). Recent reports showed that sequence type 258 (ST258) is the most prevalent clone contributing to the worldwide spread of KPC-producing *K. pneumoniae* and is the cause of numerous hospital-associated outbreaks (Kitchel et al., 2009; Hammerum et al., 2010; Marquez et al., 2014). In China, the dominant clone of KPC-producing *K. pneumoniae* is ST11 which is closely related to ST258 (Qi et al., 2011). Although many reports have identified KPC-2 producing *K. pneumoniae*, outbreak by ventilator-associated KPC-2 producing *K. pneumoniae* in China was limited. The aim of the present study was to investigate the dissemination of ventilator-associated *K. pneumoniae* with carbapenem resistance in a tertiary teaching hospital in central China. We demonstrated a outbreak by ventilator-associated ST11 *K. pneumoniae* with co-production of CTX-M-24 and KPC-2.

## Materials and Methods

### Isolation and Identification of Bacterial Strains

From January 2013 to February 2014, a total of 41 non-duplicate *K. pneumoniae* isolates with carbapenem resistance were collected, among which 28 were isolated from hospitalized patients and 13 were isolated from ventilators in a tertiary teaching hospital in Nanchang, China. The 28 isolates of CRKP were isolated from sputum (22), blood (2), ascites (1), bile (1), urine (1), and bronchoalveolar lavage fluid (1) of the patients. The first isolation of every patient was selected for further investigation. Among 28 patients infected by CRKP, 19, 3, and 2 were isolated from surgical intensive care unit (SICU), respiratory ward and gastroenterology ward, respectively. Pulmonary infection was found among 22 of 28 patients, while bloodstream infection was found in two patients. The remaining four isolates were isolated from four patients in general surgery ward, neurosurgery ward, urology ward, and nephrology ward, respectively. Twenty-four of 28 patients have been submitted to mechanical ventilation using ventilator. Consequently, 13 CRKP isolates were isolated from the ventilators used for the patients mentioned above. The patients were primarily elderly (median age of 62.5 years old, average of 23–97 years old) and subjected to multiple underlying diseases. Twenty-six of 28 patients have been submitted to multiple invasive treatment procedures, such as mechanical ventilation (24/26), venipuncture and catheterization (17/26), urinary catheterization (12/26). Moreover, all patients received multiple antimicrobials treatment in the course of hospitalization. Ventilators were sampled by rotating sterile, cotton-tipped swabs inserted into the bottom of the tubes. The bacterial isolates from sputum specimens growing more than three fourths of the plate by quantitative culture were considered to be responsible for pulmonary infection. *K. pneumoniae* isolates were identified by a Vitek-2 microbiology analyzer (bioMérieux, Marcy l’Etoile, France) in accordance with the manufacturer’s instructions. *Escherichia coli* ATCC 25922 was used as the control strain. The Ethics Committee of the first Affiliated Hospital of Wenzhou Medical University exempted this study from review because the present study focused on the bacteria.

### Antimicrobial Susceptibility Testing

Antimicrobial susceptibilities were determined initially by using Gram-negative susceptibility (GNS) cards on the Vitek system (bioMérieux, Marcy l’Etoile, France). Then multidrug resistance profiles were further evaluated by the disk diffusion test using commercial disks including minocycline (30 μg), tetracycline (30 μg), piperacillin (100 μg), piperacillin/sulbactam (110 μg), cefotaxime (30 μg), ceftazidime (30 μg), cefepime (30 μg), aztreonam (30 μg), cefoxitin (30 μg), imipenem (10 μg), meropenem (10 μg), trimethoprim/sulfamethoxazole (1.25/23.75 μg), amikacin (30 μg), gentamicin (10 μg), ciprofloxacin (5 μg), and levofloxacin (5 μg), in accordance with the criteria recommended by Clinical and Laboratory Standards Institute [CLSI] (2014). *Escherichia coli* ATCC 25922 was used as quality control strain for antimicrobial susceptibility testing.

### Detection of β-Lactamases

The carbapenemases produced by CRKP isolates were determined by modified Hodge test recommended by Clinical and Laboratory Standards Institute [CLSI] (2014). Extended spectrum beta lactamases (ESBLs) were tested among all CRKP isolates by the CLSI-recommended confirmatory double disk combination test (Clinical and Laboratory Standards Institute [CLSI], 2014).

### Investigation of Resistance Genes

The carbapenemase genes responsible for carbapenem resistance, including *bla_KPC_, *bla*_GES_, *bla*_SPM_, *bla*_IMP_, *bla*_V IM_*, *bla_SPM_*, and *bla_NDM_*, were detected using PCR and DNA sequencing as described previously (Queenan and Bush, 2007; Nordmann et al., 2011). ESBLs genes were detected in accordance with the method described previously (Andrade et al., 2010). PCR products were analyzed by electrophoresis in 1% agarose gels and were sequenced on both strands.

### Bacterial Clonal Relatedness

Pulsed-field gel electrophoresis (PFGE) was established using XbaI digestion for 4 h at 37°C and electrophoresis in agarose gels for 19 h at 14°C, 120°, with switch times of 6 and 36 s at 6 V/cm, Bio-Rad CHEF III system. Comparison of the PFGE patterns was performed with Bionumerics software (Applied Maths, Sint-Martens-Latem, Belgium) using the Dice Similarity coefficient. Clusters were defined as DNA patterns sharing more than 80% similarity. Multi-locus sequence typing (MLST) was performed with seven standard housekeeping loci (*gapA*, *infB*, *mdh*, *pgi*, *phoE*, *rpoB*, and *tonB*) as described on the *K. pneumoniae* MLST website^1^. Sequence types (STs) were identified using the online database.

## Results and Discussion

### Antimicrobial Susceptibility

All CRKP isolates showed multi-resistance to antimicrobials tested. More than 95% of the CRKP isolates were resistant to 13 antimicrobials tested, with 100% resistance to piperacillin, piperacillin/tazobactam, ceftazidime, ceftriaxone, cefotaxime, cefoperazone/sulbactam, cefepime, meropenem, and ciprofloxacin. However, the susceptibility rates of these isolates to other antimicrobials tested were high, with susceptibility rates of 95.12% to tigecycline, 92.68% to minocycline, 85.37% to tetracycline, and 85.37% to trimethoprim-sulfamethoxazole, respectively. There was similar resistance pattern between the CRKP isolates from ventilators and patients.

### Detection of β-Lactamases and Resistance Genes

**FIGURE 1:**
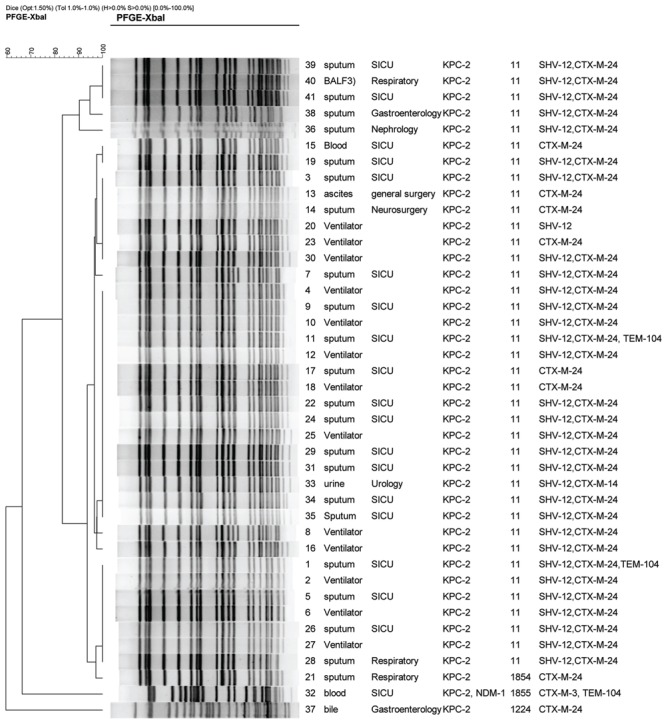
**Dendrogram of patterns for carbapenem-resistant *K. pneumonia* isolates obtained by PFGE.** SICU, surgical intensive care unit; BALF, bronchoalveolar lavage fluid; ST, sequence type; ESBLs, extended-spectrum beta-lactamases.

All CRKP isolates were confirmed as carbapenemase producer determined by the modified Hodge test. In accordance with phenotypic results of carbapenemases, all carbapenemase-producing isolates were positive for *bla*_KPC-2_, with one positive for both *bla_KPC-2_* and *bla*_NDM-1_ (**Figure 1**). The result of the present study was similar to previous reports in which *bla*_KPC-2_ was the predominant carbapenemase gene among *K. pneumoniae* in China (Chen et al., 2011; Hu et al., 2012). Other carbapenemase genes including *bla*_GES_, *bla*_IMP_, *bla*_SPM_, and *bla*_V IM_ were not detected in any of the tested isolates. Our previous report also found that 54.9%, (28/51) of carbapenem-resistant *Enterobacteriaceae* isolates expressed *bla*_KPC-2_ (Hu et al., 2014). In contrast, IMP and NDM type metallo-lactamases were also found among *K. pneumoniae* clinical isolates in China. IMP-4 was found among four *K. pneumoniae* clinical isolates from neonates and three *K. pneumonia*e isolates from the environment of neonate ICU (Yu et al., 2012). A high prevalence rate (55.0%, 22/40) of MBLs including IMP-4, IMP-8, and NDM-1 was found among CRKP isolates at a tertiary teaching hospital in southeastern China (Li et al., 2014). Co-production of different carbapenamases was also found among clinically important organisms which poses a challenge for infection control (Karthikeyan et al., 2010; Wei et al., 2011; Dortet et al., 2012; Kumarasamy and Kalyanasundaram, 2012; Rimrang et al., 2012). In the present study, coexistence of *bl*a_KPC-2_ and *bla*_NDM-1_ was found in one CRKP isolate. More type carbapenemases and co-production of different carbapenamases were found among CPKP isolates in China, which should be of concern.

Co-production of carbapenemases with other β-lactamases results in resistance to nearly all clinically available β-lactams. As carbapenemases are not inhibited by clavulanic acid, co-production of ESBLs and carbapenemases can mask identification of ESBLs by the CLSI-recommended double-disk test. Detection of multiple β-lactamases produced by *Enterobacteriaceae* in the clinical laboratory is challenging. In the present study, all carbapenemase-producing isolates harbored at least one of ESBL genes tested, among which *blaCTX*-M-24 92.7% (38/41), *bla*SHV-12 78.1% (32/41), *blaTEM*-104 7.3%, (3/41), and *blaCTX*-M-3 2.4% (1/41) harbored (**Figure 1**). Thirty-seven (85.37%) of 41 CRKP isolates were positive for narrow spectrum lactamase gene, *bla*_TEM-1_. 95.1% (39/41) of the tested isolates were found to harbor both *bla*_CTX-M-24_ and *bla*_KPC-2_, with 75.6% (31 isolates) with coexistence of *bla*_CTX-M-24_, *bla*_SHV -12_, and *bla*_KPC-2_ (**Figure 1**). In particular, two isolates with *bla*_KPC-2_ were positive for three ESBL genes including *bla*_CTX-M-24_, *bla*_SHV -12_, and *bla*_TEM-104_ (**Figure 1**). Of note, one isolate harbored simultaneously two carbapenemase genes (*bla*_KPC-2_ and *bla*_NDM-1_) and two ESBL genes (*bla*_CTX-M-3_ and *bla*_TEM-104_; **Figure 1**). *bla*_KPC-2_ has been found to coexist with various ESBL genes in one organism. However, to the best of our knowledge, coexistence of *bla*_KPC-2_ and *bla*_CTX-M-24_ in one organism is first reported.

### Bacterial Clonal Relatedness

Multi-locus sequence typing results showed that 41 CRKP isolates belonged to four STs including ST11, novel ST1854, novel ST855, and ST1224 (**Figure 1**). PFGE results displayed three PFGE clusters (**Figure 1**). Thirty-eight isolates (92.7%) including all 13 isolates from ventilators and 25 isolates from patients from six wards belonged to ST11. In accordance with MLST results, PFGE results also showed that 38 ST11 isolates belonged to same PFGE cluster with more than 80% of similarity, indicating these CRKP isolates were clonally related. Particularly, 15 isolates have an identical undistinguished pattern (100% similarity) forming a single clonal population. Moreover, this clone was exclusively linked to the cases attended in SICU and linked to the ventilators. Additionally, the other SICU cases were linked to closely related clones (similarity greater than 95%). These data indicated that the occurrence of a clonal outbreak associated with ventilators has been found.

Although the isolate harboring both *bl*a_KPC-2_ and *bla*_NDM-1_ was also from surgery ICU, it belonged to a novel ST, ST1855, which was a two-locus variant of ST11. The ST1855 isolate belonged to a specific PFGE cluster different from the PFGE cluster representing for ST11 isolates, indicating that the ST1855 isolate was not clonally related to ST11 isolates. Another novel ST, ST1854, was found in a isolate from the sputum of a patient in respiratory ward. As ST1854 was a single-locus variant of ST11, the ST1854 isolate and ST11 isolates belonged to same PFGE cluster. The ST1224 isolate belonged to another PFGE cluster. Among 25 patients infected by ST11 isolates, 18 were from surgery ICU and other seven were from five wards. ST11, a single-locus variant of ST258, was demonstrated to be the predominant ST among KPC-2 producing *K. pneumoniae* in China (Li et al., 2012). ST11 was also prevalent in Brazil (Andrade et al., 2014). Although ST258 is the epidemic clone of KPC-2 producing *K. pneumoniae* worldwide, which has been reported as dominant clone in USA (Kitchel et al., 2009), this important clone has not been detected in China. Our data indicated that the outbreak by KPC-2 -producing *K. pneumonia* has been found in SICU of this tertiary teaching hospital in central China. Other than surgery ICU, sparse ST11 isolates belonging to same PFGE cluster were found in general surgery ward, Neurosurgery ward, Urology ward, and Nephrology ward, indicating KPC-2 -producing ST11 *K. pneumoniae* clone has disseminated at this hospital. It is of concern that outbreak by this important clone will occurred in other wards. Pulmonary infection caused by ST11 CRKP was found among18 patients from surgery ICU who were submitted to mechanical ventilation using the ventilators contaminated by KPC-2 -producing *K. pneumoniae*. Moreover, the CRKP isolates from patients from surgery ICU were clonally related to the isolates from ventilators. Therefore, we speculated that the outbreak by KPC-2 -producing ST11 *K. pneumoniae* clone was associated with ventilators. To control the spread of carbapenemase-producing organism in hospital, strict infection control measures such as reinforcement of hand hygiene, contact precautions, disinfection of equipments, disinfection of contaminated environment around patients should be carried out.

## Conclusion

An outbreak caused by ventilator-associated ST11 *K. pneumoniae* with co-production of CTX-M-24 and KPC-2 has been found in a surgery ICU of a tertiary teaching hospital in central China.

## Author Contributions

LH, YL, LD, QZ, YH, ZW, and LZ performed the laboratory measurements. FY and LW made substantial contributions to conception and design. FY and LW revised the manuscript critically for important intellectual content. YL, LH, and FY participated in experimental design and data analysis. FY drafted the manuscript. All authors read and approved the final manuscript.

## Conflict of Interest Statement

The authors declare that the research was conducted in the absence of any commercial or financial relationships that could be construed as a potential conflict of interest.

## Footnotes

1 http://www.pasteur.fr/recherche/genopole/PF8/mlst/Kpneumoniae.html
